# Metastatic differentiated thyroid cancer: worst prognosis in patients with metachronous metastases

**DOI:** 10.1007/s12020-023-03302-0

**Published:** 2023-05-12

**Authors:** Angélica María González-Clavijo, Andrés A. Cuellar, Jenny Triana-Urrego, Jorge A. Barrero, Luis Felipe Fierro-Maya

**Affiliations:** 1 Endocrine Oncology Unit, Instituto Nacional de Cancerología, Bogotá, Colombia; 2 Department of Physiological Sciences, Faculty of Medicine, Universidad Nacional de Colombia, Bogotá, Colombia

**Keywords:** Metastases, Progression, Radioiodine, Thyroid cancer

## Abstract

**Purpose:**

To describe the overall survival and progression-free survival in patients diagnosed with differentiated thyroid carcinoma with synchronous and metachronous metastatic involvement.

**Materials and methods:**

A retrospective cohort study was conducted with 101 patients with differentiated thyroid cancer (DTC) who had metastatic involvement at diagnosis or during follow-up, who were treated at the National Cancer Institute between January 1, 2010 and December 31 of 2015.

**Results:**

81 patients (80.2%) were women and the mean age at diagnosis was 49 years (12–80). Synchronous metastases were detected in 54.5% of patients and metachronous metastasis was diagnosed in 45.5% of patients, in whom the mean time between initial diagnosis and the finding of distant metastases was 5 years. Pulmonary involvement occurred in almost all patients, with ^131^I uptake in 58% of synchronous metastases and in 21% of metachronous. There were 10 events in the patients with ^131^I-avid metastases with a median time to progression that was not reached, and there were 23 events in patients with ^131^I-refractory metastases with a median time to progression of 96 months; The median time to progression was significantly longer in patients with synchronous metastases compared to those with metachronous metastases (Not reached vs 95 months, *P* = 0.017) The 5-year overall survival rate was 95% to the entire cohort.

**Conclusions:**

The present study contributes to the expansion of the knowledge about this clinical course of DTC with the finding of a worst prognosis in patients with metachronous metastases.

## Introduction

Differentiated thyroid cancer (DTC), comprising papillary and follicular carcinoma, is the most common malignant endocrine neoplasm in adults and accounts for more than 90% of all thyroid cancers [1]. Distant metastasis occurs in around 6–23% of patients with DTC [2–5], and while radioiodine (RAI) ablation therapy is the first-line treatment in metastatic disease, one-third of patients either do not uptake ^131^I or become RAI-refractory [3, 6]. In the latter group, alternative treatments such as multikinase inhibitors and targeted therapies for tumors with specific molecular alterations have primarily resulted in time to progression prolongation [7–10]. Since almost one-third of patients with metastatic compromise may remain stable for up to 10 or more years [3, 11], active surveillance is a rational approach to patient care while limiting surgical or pharmacological interventions to those with progressive disease or mass effect symptoms.

A retrospective study was designed to describe the overall survival and progression-free survival in patients diagnosed with differentiated thyroid carcinoma with synchronous and metachronous metastatic involvement who were treated at the National Cancer Institute between January 1, 2010, and December 31, 2015.

## Materials and methods

### Study design and population

A cohort study was conducted with a retrospective review of the patient registries of the multidisciplinary thyroid cancer board of the Colombian National Cancer Institute (INC) to identify cases who had been treated between January 1, 2010, and December 31, 2015. Some patients were diagnosed before January 2010 but were included given that they received medical, surgical or, RAI therapy during the study period. Patients who met the following inclusion criteria were selected: >18 years old with histological diagnosis of differentiated thyroid cancer with extra cervical metastasis detected at the time of diagnosis, in the post-surgical stratification, or during follow-up. Subjects with poorly differentiated carcinoma, anaplastic carcinoma, and medullary thyroid carcinoma, as well as those with loss to follow-up for more than 2 consecutive years were excluded. Data were collected by reviewing the patients’ clinical records in a systematized database (RedCap(R)).

The institutional protocol of care of patients with thyroid cancer includes the discussion of all cases in a multidisciplinary board approximately 6 weeks after the surgical intervention for cases operated on in the institution or after an initial endocrinology consultation for cases operated in other institutions. Computed tomography images of the thorax are routinely performed to all patients with histological or clinical criteria of high-risk category ATA 2015, and to patients with histological angioinvasion. Patients in the low-risk category for relapse do not receive iodine ablation unless they have elevated thyroglobulin or anti-thyroglobulin antibodies; those with intermediate risk for relapse receive a dose between 30 and 100 mci, at the discretion of the multidisciplinary board, and those with high risk receive an activity of 100 mci when there is no evidence of metastasis and 200 mci, for those with suspected metastasis by CT imaging. All patients who received an ablative or therapeutic dose of ^131^I underwent a whole-body scan and SPECT CT one week later. During follow-up, Chest CT images are performed in those patients with incomplete biochemical response and those with known metastatic disease to assess response. 18F-FDG PET is performed in patients with incomplete biochemical response without detectable disease by neck ultrasound and chest tomography. Magnetic resonance imaging (MRI) or CT of other organs is performed in those patients with symptoms suggestive of compromise or due to abnormal uptakes in the post therapy scan. Tumor responses were classified as Complete, when meet the criteria of excellent biochemical and structural response. Patients with incomplete structural response were divided according to RECIST 1.1 criteria in partial response (at least a 30% decrease in the sum of the longest diameter of measures target lesions, taking as reference the baseline sum of the longest diameters; progressive disease (increase of 20% or more in the sum of the longest diameters); and Stable disease (Neither sufficient shrinkage to qualify for partial response nor sufficient increase to qualify for progressive disease).

### Statistical analysis

Univariate analyses were performed by calculating measures of central tendency and dispersion according to the distribution characteristics of each of the quantitative variables, while the categorical variables were presented in absolute and relative frequencies. Bivariate analysis of the association between clinical and histologic variables and regression output was estimated using the chi-square or Fisher’s exact test for categorical variables, and the Mann-Whitney test or the t-test for continuous variables, with the calculation of the relative risk (RR) values. The survival function was determined under the assumption of proportionality using the Kaplan-Meier method. Values were calculated with a 95% confidence interval and the statistical analysis was performed in the SPSS statistical package version 26 (z125-330114).

## Results

Between January 1, 2010 and December 31, 2015, a total of 2620 new cases of CDT were treated in our institution, and 53 patients of them had metastatic involvement at diagnosis or during follow-up, and met the inclusion criteria. Additionally, 48 patients diagnosed between 2005 and 2009 with metastatic disease were included, because they received some treatment for metastases during the study period. For the total population, 81 patients (80.2%) were women and the mean age at diagnosis was 49 years (12–80). The mean follow-up time from the time of diagnosis was 122 months (48–375).

The main characteristics of the patients included in the analysis are shown in Table 1. The mean dominant primary tumor diameter in the thyroid gland was 3.1 cm (0.3–7.9) and the most common histologic subtype of DTC was papillary carcinoma, found in 90 patients (89.1%), followed by follicular carcinoma in 10 patients (9.9%) and 1 patient with Hürtle cell carcinoma. The most frequently found variants of papillary thyroid carcinoma were classical (67.3%) and follicular (12.8%). Cervical lymph node compromise at diagnosis was evident in 86 patients (85%) of which 52 had involvement in both central and lateral compartments, and extra nodal extension was found in 21 patients. All patients were treated with total thyroidectomy and 75 patients (74.2%) received an initial dose of radioiodine between the 2- and 12-month post-thyroidectomy period.

**Table 1:** Demographic and histologic characteristics of patients with DTC

Variable	
Mean age (range) years	49 (12–80)
Sex	
Women (*n* (%)	81 (80.2)
Men (*n* (%)	20 (19.8)
Mean tumor diameter (range) cm	3.1 (0.3–7.9)
Papillary Thyroid carcinoma *n* (%)	90 (89.1)
Follicular Thyroid carcinoma *n* (%)	10 (9.9)
Hürtle cell carcinoma *n* (%)	1 (0.9)
Cervical lymph node compromise at diagnosis *n* (%)	73 (72.2)

Synchronous metastatic disease, defined as the finding of distant metastases in perioperative staging studies up to 6 months after surgery or in the first radioiodine body scan, was evident in 55 patients. Out of these, 52 patients had exclusive pulmonary metastatic compromise, 2 patients had an exclusive bone metastasis, and 1 patient had both pulmonary and bone metastatic compromise.

On the other hand, metachronous metastasis was diagnosed in 46 patients, all with pulmonary compromise and only 8 patients with additional metastases in other sites (four to the central nervous system and four to the liver). The mean time between initial diagnosis and the finding of distant metastases was 5 years (0.7–21). Both groups of patients had elevated mean pre-ablative thyroglobulin levels (Synchronous: 1148 ng/ml and Metachronous: 1021 ng/mL) without statistical differences (*P* = 0.68).

Overall, distant ^131^I-avid metastases accounted for 42 patients (41.5%). The rate of patients with ^131^I avidity was 58.1% (32/55) in those with synchronous metastases, and 21.7% (10/46) in those with metachronous metastases, with a non-significant difference (*P* = 0.36). Table 2 summarizes the comparison between both groups of patients.

**Table 2:** Comparison between patients with synchronous and metachronous metastases

TNM AJCC 8th	Synchronous	%	Metachronous	%	*p*
Tx	9	16.4	15	32.6	0.056
T1a	1	1.8	2	4.3	0.455
T1b	11	20.0	7	15.2	0.531
T2	17	30.9	12	26.1	0.593
T3a	5	9.1	4	8.7	0.944
T3b	9	16.4	3	6.5	0.127
T4a	3	5.5	2	4.3	0.798
T4b	0	0.0	1	2.2	NA
Nx	4	7.3	12	26.1	0.0099
N0	4	7.3	8	17.4	0.117
N1a	29	52.7	8	17.4	0.0002
N1b	18	32.7	18	39.1	0.503
Mx	0	0.0	9	19.6	NA
M0	0	0.0	37	80.4	NA
M1	55	100.0	0	0.0	NA
Metastasis site					
Lung	53	96.4	46	100	
Bone	3	5.5	0	0.0	
CNS	0		4	8.7	
Liver	0		4	8.7	
^131^I-Avid metastasis *n*, %	32	58.1	10	21.7	0.36
Mean cumulative ^131^I dose (range) mCi	361 (100–920)		405 (100–900)		
Mean pre-ablation Thyroglobulin (range) ng/ml^a^	1148.7 (2.0–9223)		1021.5 (2.0–7246)		0.68
Radiation therapy (Any site)	10	18.2	8	17.4	
Treatment with tyrosine kinase inhibitors *n*, %	8	14.5	7	15.2	
Disease status at the last visit					
Partial Response *n* %	7	12.7	1	2.2	0.005
Estable disease *n* %	33	60	31	67.4	0.442
Progression *n* %	7	12.7	11	23.9	0.143
Deaths *n* %	8	14.5	3	6.5	0.197

^a^Data valid only in patients without antithyroglobulin antibodies interference.

**Fig. 1:**
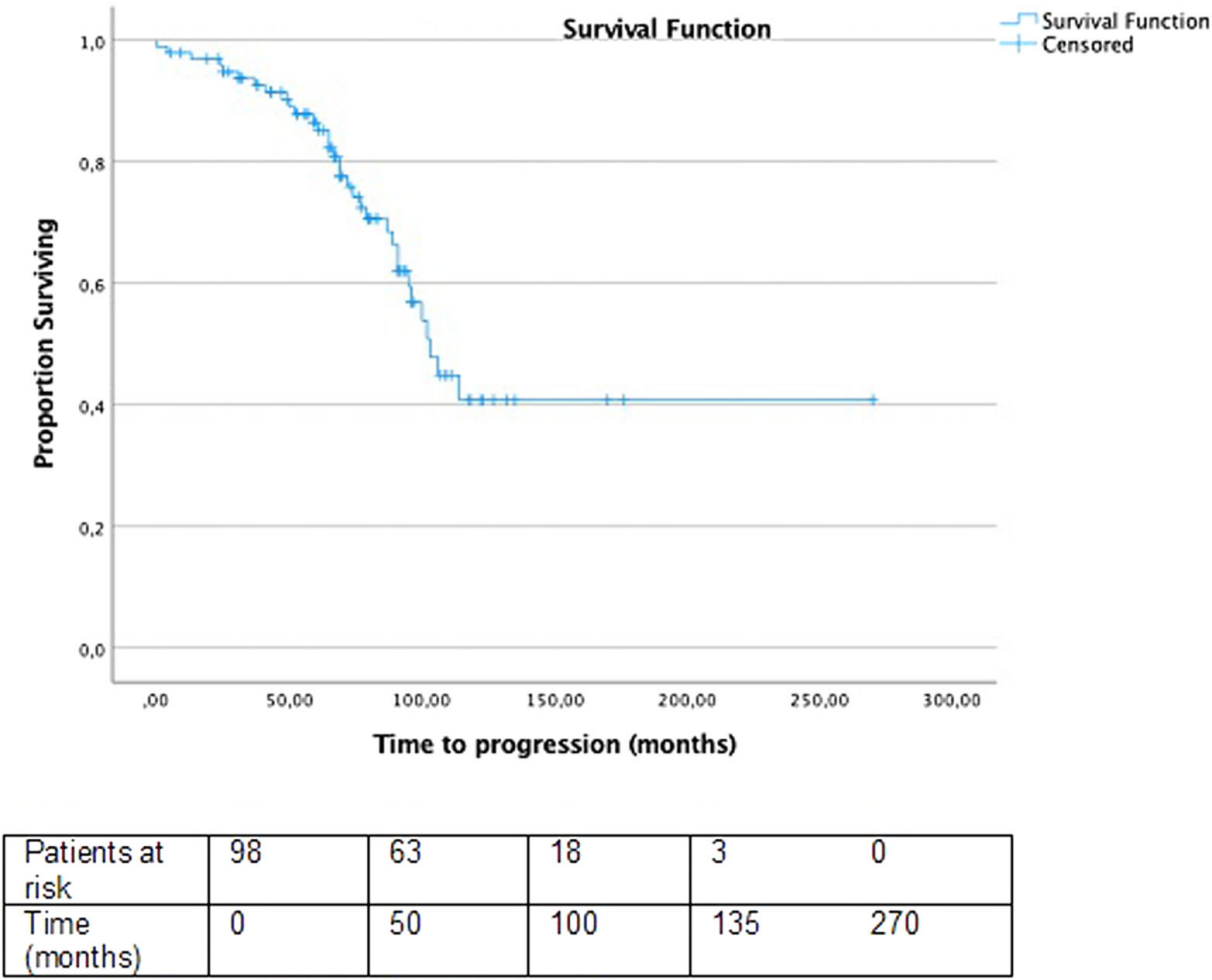
Progression-free survival of patients. Median: 103 months Events: 33 Censored: 66

**Fig. 2:**
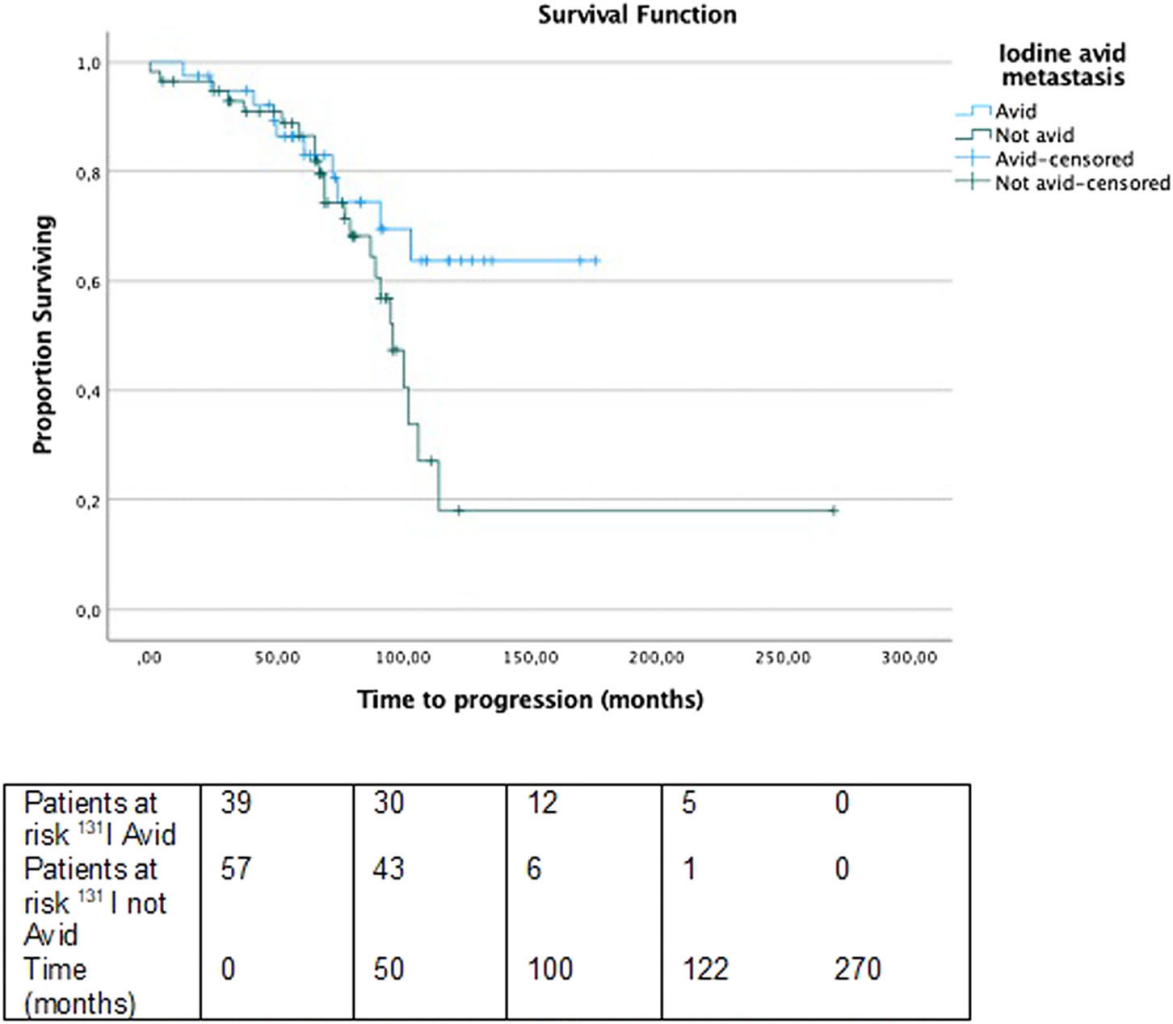
Progression-free survival stratified by ^131^ I uptake. Median time to progression in patients with 131 I-avid metastates: not reached; events: 10,censored: 40. Median time to progression in patients with 131 I-refractory metastatis: 96 months; events: 23, censored: 35. Log-rank test, P = 0.057

**Fig. 3:**
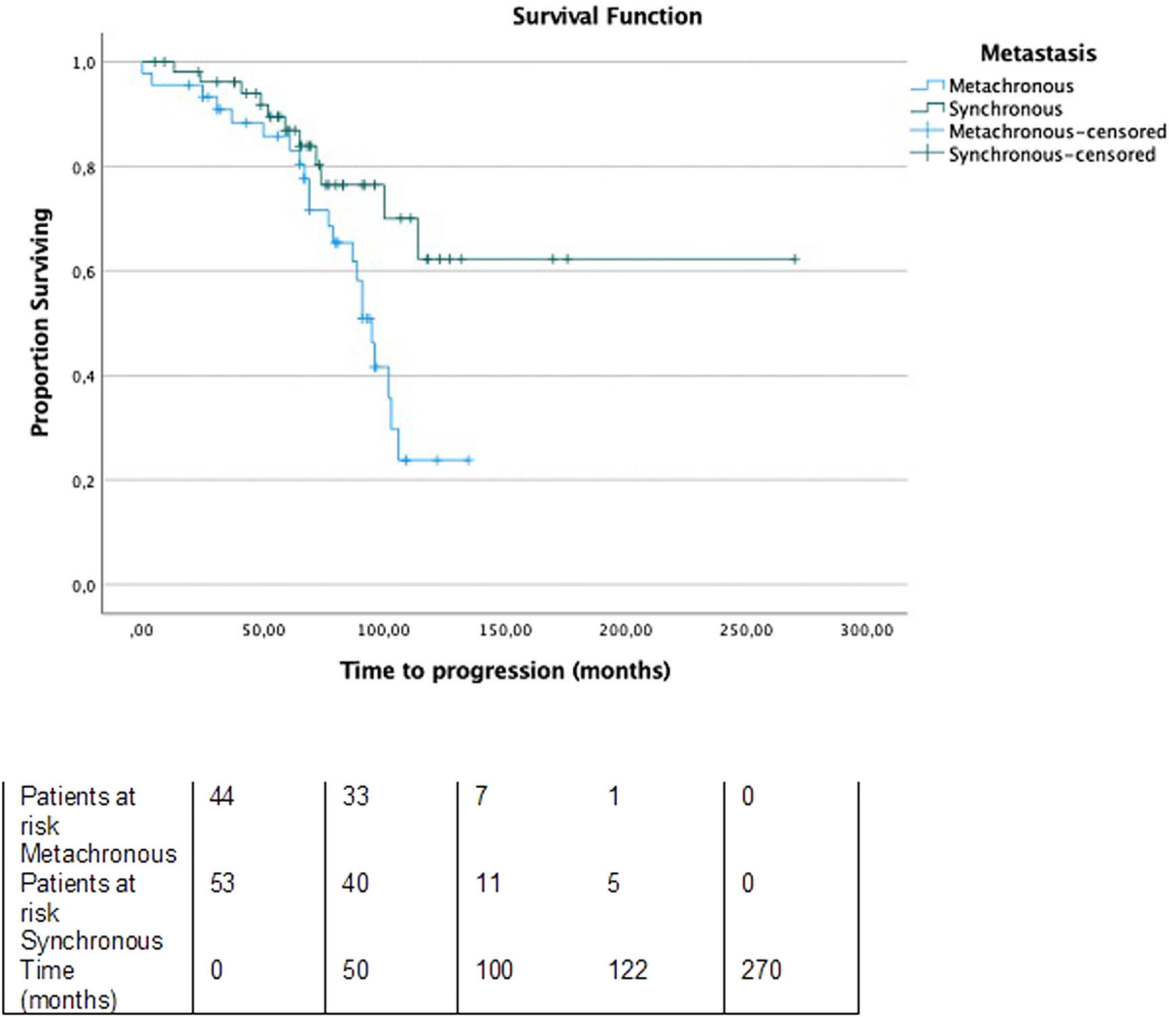
Progression-free survival stratified by the time of metastasis diagnosis (synchronous vs metachronous). Median time to progressionin patients with synchronous metastates: not reached; events: 11, censored: 43. Median time to progression in patients with metachronousmetastates: 95 months; events: 22, censored: 23. Log-rank test, P = 0.017

The mean time to progression, estimated between the date of metastasis diagnosis and the finding of progression (determined by the growth of 20% or more in the sum of the diameters of the target lesions, or by new lesions) using computed tomography imaging, was 103 months as shown in Fig. 1. In patients with ^131^I-refractory metastases, the mean time to progression was 96 months while in patients with ^131^I-avid metastases it was not reached (*P* = 0.057) (Fig. 2). The median time to progression was significantly longer in patients with synchronous metastases compared to those with metachronous metastases (Not reached versus 95 months, *P* = 0.017) (Fig. 3).

A total of 15 patients received Sorafenib after being considered as iodine refractory, 8 with synchronous metastases and 7 with metachronous metastases. The average time of use of Sorafenib was 13.2 months (range 1–28) Three patients reached partial response, and 11 patients had stable disease for 6 or more months; One patient discontinued treatment due to toxicity.

The disease status at the time of last visit is summarized in Table 2. There were more partial responses in patients with synchronous metastases (*p* 0.005). We did not find complete responses (structural excellent response).

**Fig. 4:**
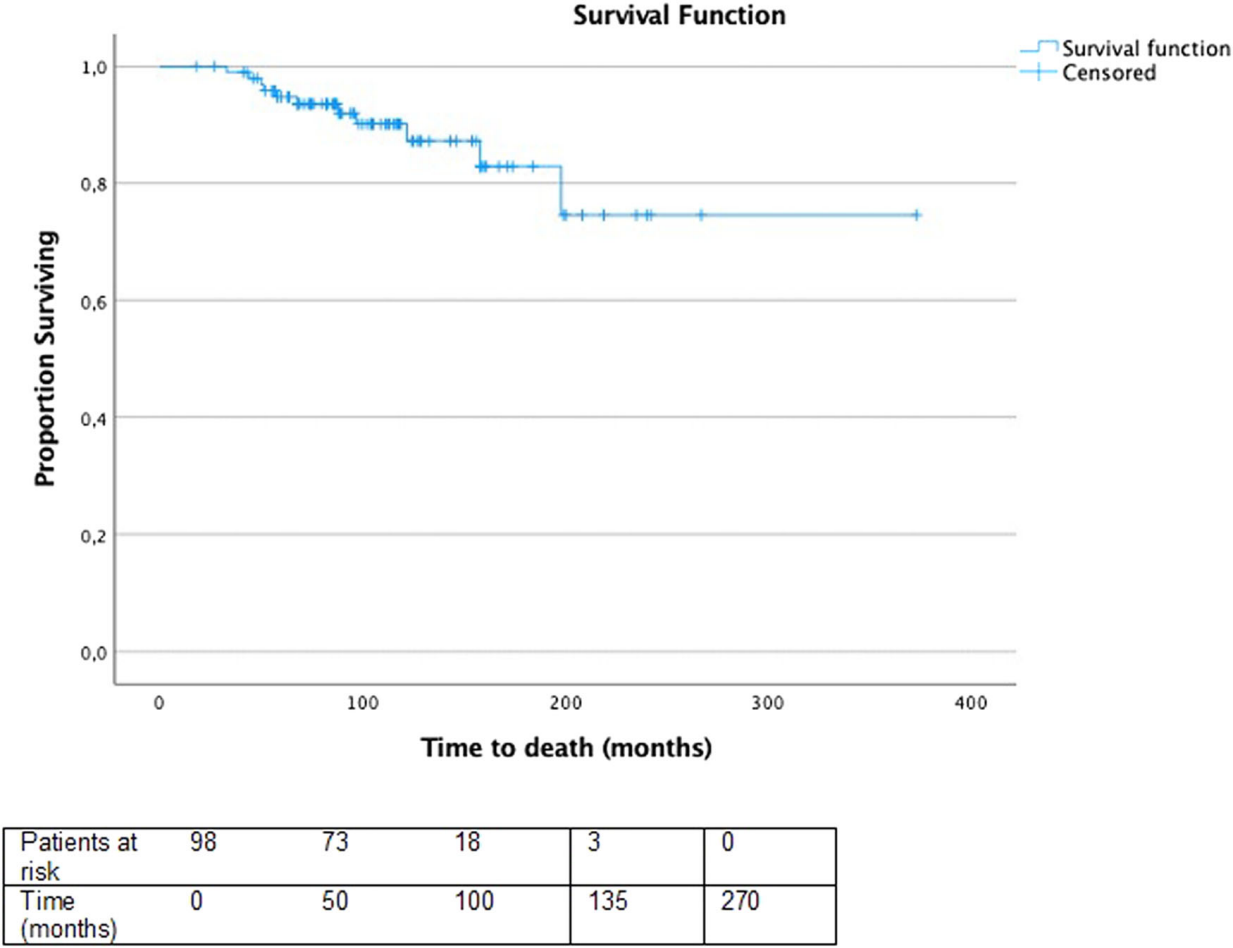
Overall survival. Median survival: not reached; events: 11, censored: 90

The bivariate analysis of qualitative variables showed that patients with synchronous metastases had a lower risk of progression, with a RR value of 0.496 (95% CI: 0.294–0.825; *P* < 0.05). There were 11 deaths during the follow-up time, 8 of 55 patients with synchronous metastases (14.5%) and 3 of 46 patients with metachronous metastases (6.5%) died in the follow-up period. Among the 11 patients who died, 6 received Sorafenib, with an average duration of treatment of 7.4 months. In the entire cohort, the median overall survival was not reached (Fig. 4). The 5-year overall survival rate was 95%.

## Discussion

We present the baseline characteristics and the evolution of a cohort of 101 patients with DTC who had metastatic involvement at diagnosis or during follow-up. Synchronous metastasis was found in 54.5% of patients, and pulmonary involvement occurred in more than 90% of the whole cohort. The mean time to progression, estimated between the date of metastasis diagnosis and the finding of progression was 103 months and the overall survival rate was 95% at 5 years. Our results are similar to those found in other retrospective studies [3, 11–13], emphasizing the concept of a not-so-aggressive course of metastatic disease in DTC, with a latency time of the disease that confers a better prognosis, compared to that observed in other studies.

Ringel et al. [14] hypothesize that most recurrences in distant sites account for the growth of pre-existing metastases that were previously inactive, below the detection threshold of diagnostic imaging (biological and clinical dormancy), so early diagnosis of clinically asymptomatic distant metastases is not simple, but the implementation of the risk stratification of recurrence in thyroid cancer established in the American DTC management guidelines [1] enables clinicians to identify more precisely which patients will undergo complementary studies in an effort to promptly diagnose metastases. One of these criteria corresponds to elevated thyroglobulin levels following thyroidectomy surgery, and there are several retrospective studies with different cut-off points [15–17]. In our study, the mean pre-ablation thyroglobulin was considerably elevated in both synchronous and metachronous metastases, with a rather long latency time for patients with metachronous metastatic disease, with a mean time between initial diagnosis and the finding of distant metastases of 5 years, similar to that found in the study by Sabet et al. [12].

The prognosis in terms of progression-free survival was better for patients with synchronous metastases in our cohort, similar to that reported by Sabet et al. [12] and Kim et al. [18]. Speculatively, it is possible that during this long clinical latency period, cancer cells become dedifferentiated, as we found a lower proportion of patients with ^131^I avid metastases in the metachronous metastasis group and, as is well known, the prognosis is worse for patients with iodine-refractory metastases, as confirmed in our study, with a shorter time to progression in iodine-refractory metastases, similar to that found in other series [3, 11, 13]. In the same sense, there were more partial responses in the group of patients with synchronous metastases (*p* = 0.005).

It is important to highlight that 8 patients with metachronous metastases had involvement in organs such as the CNS and liver, which have a worse prognosis, but no patient with synchronous metastases had involvement in those sites.

## Conclusions

The present study contributes to the expansion of the knowledge about the clinical course of patients with metastatic DTC, in special with regard to differences in the outcomes in patients with synchronous and metachronous metastases, with a poor prognosis in the latter group, which should alert us to the need to improve search strategies to early detect the metastatic involvement, especially in patients with incomplete biochemical responses, and provide more timely treatment.
